# Unilateral Uterine and Ovarian Arterial Ligations in a Case of Missed Abortion at 12 Weeks of Gestation with Undiagnosed Placenta Accreta

**DOI:** 10.7759/cureus.3562

**Published:** 2018-11-08

**Authors:** Fatimah Alnafisah, Sherif Abdelaty Alalfy

**Affiliations:** 1 Obstetrics & Gynecology, King Saud Hospital, Unizah, SAU

**Keywords:** placenta accrete, first trimester, dilatation and evacuation

## Abstract

Placenta accreta is abnormal placental attachment to the myometrium, and the incidence rate has risen with the increased use of Cesarean sections. First-trimester placenta accreta is a rare, potentially life-threatening condition due to the severe hemorrhage it may cause, necessitating a hysterectomy. We present a case of a 38-year-old woman with a history of two Cesarean section deliveries who developed severe bleeding during curetting due to undiagnosed placenta accreta. Unilateral uterine and ovarian arterial ligations were performed to reduce expected bleeding along with a local resection of the placental implantation site that was invading the old scar. This procedure was effective with fewer complications than traditional procedures and preserved the patient’s fertility.

## Introduction

Undiagnosed placenta accreta in early pregnancy is a life-threatening condition and can lead to massive hemorrhage. Previous Cesarean sections increase the risk of placental abnormalities [1]. Early detection of these abnormalities can lead to a clear management plan to prevent maternal morbidity and mortality. In most reported cases, hysterectomy was the main option to stop the severe bleeding [2]. Uterine and ovarian arterial ligations are sometimes necessary to save the uterus in women early in their reproductive period. This case is an example of such a success.

## Case presentation

**Figure 1:**
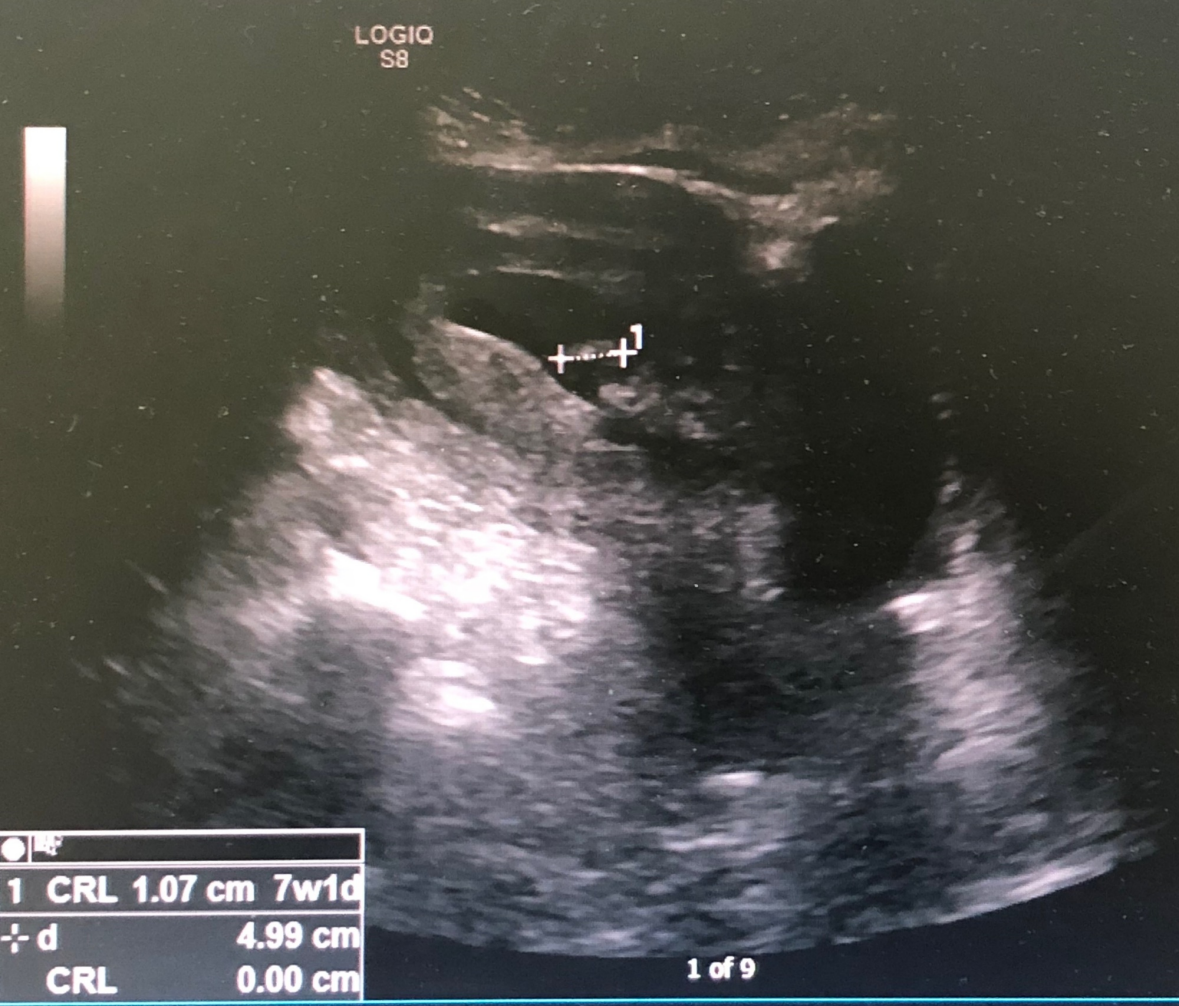
Ultrasound showing an intrauterine single nonviable fetus with a crown-rump length corresponding to seven weeks

A 38-year-old woman (gravida 4, para 2, abortus 1) at 12 weeks of gestational age was admitted as a case of missed abortion. Ultrasound (US) showed an intrauterine single nonviable fetus with a crown-rump length corresponding to seven weeks (Figure 1).

The patient refused medical termination and preferred expectant management to wait for a spontaneous abortion. After two weeks, the patient presented back at the emergency department describing the new onset of mild vaginal bleeding. She had undergone two previous Cesarean deliveries; the most recent Cesarean was six years prior to this presentation to the emergency department. Other previous surgical procedures included an appendectomy and a partial gastrectomy.

The patient was admitted with minimal vaginal bleeding and mild lower abdominal pain. Her vital signs were stable, and her cervical os was closed during per vaginal (PV) examination. 

The patient was treated with misoprostol PV. After three hours, she developed severe bleeding. A PV examination revealed the os was tip-of-finger, and a speculum examination revealed no cervical laceration. At this time, the patient was passing blood clots. Bedside US showed a bulky uterus containing retained products of conception and a uterine cavity filled with blood clots.

The patient was moved to the operating theater for evacuation and curettage (E&C). Uterine content and blood clots were evacuated by ovum forceps, but E&C failed to stop the current bleeding situation. A laparotomy was performed, and the abdomen was opened via a Pfannenstiel incision. Her uterus was intact, and no tear or perforation was noted. A diagnosis of placenta accreta was made intraoperatively. The placenta was in the lower uterine segment invading the old scar. Unilateral ligation of the uterine and ovarian arteries was performed, along with a local resection of the uterine wall segment affected by the placenta accreta. The uterine wall defect was repaired. This procedure succeeded in stopping the bleeding and a need for a hysterectomy was avoided. The patient received five units of packed red blood cells intraoperatively and four units of fresh frozen plasma. Following the procedure, she received an intramuscular injection of methotrexate 50 mg as an adjuvant therapy along with an antibiotic and anticoagulant. The patient was discharged five days following the operative procedure without sustaining any noted complications.

## Discussion

A first-trimester hemorrhage after an abortion is rare (<1% occurrence). The occurrence of hemorrhage in early pregnancy may be due to uterine atony, abnormal placentation, and coagulopathy. Furthermore, hemorrhage can occur as a complication following a procedure (e.g., perforation, cervical laceration, and retained products) [1]. Abnormal placentation that invades into and beyond the myometrium can also lead to severe hemorrhage.

Placenta accreta is mainly seen in women with a previous uterine scar. The incidence of placenta accreta has increased four-fold recently due to an increase in the number of Cesarean section deliveries, occurring in approximately three in 1,000 deliveries [1]. Two large cohort studies found that approximately 1% to 2% of patients may have bleeding after a surgical abortion that will require a follow-up visit or secondary surgical intervention [1,3-4].

During the first trimester, diagnosing placental abnormalities is very difficult and has a low accuracy, compared to a diagnosis during the third-trimester [5]. Usually, placenta accreta is diagnosed after profuse hemorrhage during curettage, like in our case, or in the first week following abortion [6]. Hemorrhaging usually occurs at the time of placental detachment or during the removal of placental tissues from abnormal implantation sites, which will require immediate resuscitation. Undiagnosed placenta accreta with first-trimester abortions are common because placenta accreta rarely leads to severe hemorrhage in early pregnancy [1]. Placental localization should be confirmed in all women with a previous uterine scar.

Stirnemann et al. investigated the early detection of placenta accreta in high-risk patients in order to improve outcomes and prevent complications. They conducted a prospective US screening trial in high-risk patients (i.e., those with a uterine scar) at 11 to 14 weeks of gestational age. Using the uterine scar as a potential risk factor enabled early diagnosis and a subsequent optimal management plan to prevent massive hemorrhage and improve maternal outcomes [7].

In the event of hemorrhage during abortion or curetting, a detailed examination should be conducted to detect cervical lacerations, uterine atony, and any retained tissues. Abnormal placentation should be suspected especially in patients with a previous uterine scar. Hematological abnormalities should be excluded by laboratory evaluation [1].

Most cases of placenta accreta during the first trimester are diagnosed either after the occurrence of severe bleeding, during curettage or after abortion. Shaamash et al. summarized 23 cases in which placenta accreta was discovered during curettage where abnormal placentation was not suspected [2].

US is the main imaging modality for the evaluation of placenta accreta given the fact that color Doppler improves the ability to detect placenta accreta [8]. Sonographic findings of placenta accreta in the first trimester are described as a low-lying gestational sac with diffuse dilatation of the intraplacental lacunae in the lower uterine corpus [5].

The following three studies describe findings of intraplacental lacunae and present useful information for diagnosing placenta accreta in the first trimester of pregnancy [5]. The first is a retrospective study that described the sonographic characteristics of placenta accreta in the prenatal period in patients with a history of Cesarean delivery. The US examination was done before 10 weeks of gestation. All of these patients demonstrated a gestational sac located in the lower uterine segment at the site of the Cesarean section scar, and histopathology examination confirmed the diagnosis of placenta accreta in all of these patients [5,9]. Magnetic resonance imaging is indicated if US findings are insufficient or to localize the extension of the placental invasion.

In the next publication, placenta accreta was diagnosed by US at nine weeks of gestation, which means that the early diagnosis of placenta accreta in the first trimester is possible. Placental adhesion was diagnosed on two-dimensional sonography and color Doppler imaging by detecting the placental lacunae, which is another finding in the diagnosis of an adherent placenta. One patient developed bleeding at 15 weeks of gestation, which was managed by hysterectomy [10]. In another case report, a patient presented at eight weeks of gestation with diffuse dilatation of the subplacental vessels in the lower uterine corpus as noted by power Doppler imaging. Placenta previa/accreta was confirmed at 15 weeks of gestation by color Doppler imaging which showed Grade 3+ lacunae, and a subsequent hysterectomy was performed due to ongoing hemorrhaging [11].

Lastly, Yang et al. reported in a case report that a patient had presented at 12 weeks of gestation and was diagnosed with placenta accreta by color Doppler imaging showing large irregular lacunae with dilated intraplacental vessels. This patient’s condition was managed by bilateral uterine arterial embolization to decrease the expected bleeding during hysterectomy [5].

In general, the management of hemorrhage is resuscitation first, if the patient is not stable. Then a thorough examination is conducted to find the source of bleeding. In cases of placenta accreta, the majority of patients are ultimately treated with hysterectomy [2].

Uterine artery embolization is one of the main options for decreasing bleeding prior to considering a hysterectomy [5]. Another conservative procedure described in recent reports is to try to preserve fertility by using either laparoscopic or surgical resection of the affected part of the uterus with angiographic uterine arterial embolization [12]. Keeping the placenta in situ and administering methotrexate as adjuvant therapy for placental resorption is another option [2,13].

Shehata et al. reviewed different uterine sparing techniques in the management of placenta accreta which might have fewer complications and shorter operative times compared with conservative treatments that keep the placenta in situ. These techniques include resection of the placental implantation site or compression sutures with pelvic devascularization or a combination of both [14].

Our case was managed by unilateral uterine and ovarian arterial ligations to reduce the expected bleeding followed by local resection of the placental implantation site. Methotrexate was given as adjuvant therapy. Salvat et al. reported a 100% success rate using a stepwise procedure with progressive ligation of the uterine and ovarian arteries compared to a 66% success rate in cases with bilateral ligation of the hypogastric artery [14-15].

Gungor et al. indicate that a stepwise procedure with progressive ligation of the uterine and ovarian arteries has a 100% success rate, and they recommend ligation of the uterine and ovarian arteries first rather than an internal iliac arterial ligation due to improved efficacy and a lower level of procedural difficulty [14,16].

Placenta accreta can be safely treated by local resection of the placental implantation site and repair of the uterine defect. This procedure provides immediate management with less blood loss and preserves patient fertility [14]. This method also offers fewer complications than a hysterectomy or expectant management that leaves the placenta in situ [17].

## Conclusions

Undiagnosed placenta accreta within the first-trimester abortion is common, because placenta accreta rarely leads to severe hemorrhage early in a pregnancy. Placental localization should be confirmed in all women with a uterine scar. Unilateral uterine and ovarian arterial ligation with resection of the placental implantation site and repair of the uterine defect are options for treating placenta accreta. This procedure is effective with fewer complications and preserves the patient’s fertility.
